# Clinical applications of adipose-derived stromal vascular fraction in veterinary practice

**DOI:** 10.1080/01652176.2022.2102688

**Published:** 2022-08-08

**Authors:** Khan Sharun, Kaveri Jambagi, Rohit Kumar, Mudasir Bashir Gugjoo, Abhijit M. Pawde, Hardeep Singh Tuli, Kuldeep Dhama, Amarpal

**Affiliations:** a Division of Surgery, ICAR-Indian Veterinary Research Institute, Bareilly, Uttar Pradesh, India; b Division of Medicine, ICAR-Indian Veterinary Research Institute, Bareilly, Uttar Pradesh, India; c Faculty of Veterinary Sciences & Animal Husbandry, SKUAST-Kashmir, Division of Veterinary Clinical Complex, Srinagar, Jammu and Kashmir, India; d Department of Biotechnology, Maharishi Markandeshwar (Deemed to be University), Ambala, Haryana, India; e Division of Pathology, ICAR-Indian Veterinary Research Institute, Bareilly, Uttar Pradesh, India

**Keywords:** Mesenchymal stem cells, stromal vascular fraction, bone healing, regenerative medicine, enzymatic digestion, adipose tissue, veterinary medicine, review

## Abstract

Adipose tissue-derived stromal vascular fraction (AdSVF) comprises a heterogeneous cell population, including the multipotent mesenchymal stem cells, hematopoietic stem cells, immune cells, endothelial cells, fibroblasts, and pericytes. As such, multipotent adipose tissue-derived mesenchymal stem cells (AdMSCs), are one of the important components of AdSVF. Commonly used techniques to harvest AdSVF involve enzymatic or non-enzymatic methods. The enzymatic method is considered to be the gold standard technique due to its higher yield. The cellular components of AdSVF can be resuspended in normal saline, platelet-rich plasma, or phosphate-buffered saline to produce a ready-to-use solution. Freshly isolated AdSVF has exhibited promising osteogenic and vasculogenic capacity. AdSVF has already been proven to possess therapeutic potential for osteoarthritis management. It is also an attractive therapeutic option for enhancing wound healing. In addition, the combined use of AdSVF and platelet-rich plasma has an additive stimulatory effect in accelerating wound healing and can be considered an alternative to AdMSC treatment. It is also widely used for managing various orthopaedic conditions in clinical settings and has the potential for regenerating bone, cartilage, and tendons. Autologous AdSVF cells are used along with bone substitutes and other biological factors as an alternative to conventional bone grafting techniques owing to their promising osteogenic and vasculogenic capacity. It can also be used for treating osteonecrosis, meniscus tear, chondromalacia, and tendon injuries in veterinary practice. It has several advantages over *in vitro* expanded AdMSC, including precluding the need for culturing, reduced risk of cell contamination, and cost-effectiveness, making it ideal for clinical use.

## 1. Introduction

The adipose tissue is a multifunctional tissue that acts as an energy storehouse and plays an essential role in endocrine and immune responses. The adipose tissue contains several cell types in addition to the mature adipocytes that are embedded into an extracellular matrix (Marx et al. 2015). Fat can either be surgically extracted or liposuctioned to obtain the lipoaspirate. The mechanical or enzymatic digestion of fragmented adipose tissue releases the cellular constituents from the extracellular matrix (Marx et al. 2015; Si et al. 2019). This mixture of various cell types is known as the adipose-derived stromal vascular fraction (AdSVF). AdSVF comprises heterogeneous cell populations that include adipocytes, pericytes, endothelial cells, pre-adipocytes, and various other cells, including stem cells (Bourin et al., 2013; Lee et al. 2013; Si et al. 2019). Although fat-derived mesenchymal cells have been studied for several decades, in 2001, a significant finding was reported that a stem cell population of mesenchyme origin exists in lipoaspirate that could be isolated and maintained *in-vitro* for extended periods (Zuk et al. 2001; 2002). Furthermore, a study in the following year demonstrated their ability to differentiate into neural-like cells (Safford et al. 2002). Subsequently, their transdifferentiation potential was extended to numerous other cell lineages. These cells, known as multipotent adipose tissue-derived mesenchymal stem cells (AdMSCs), are one of the important components of AdSVF (Kim et al. 2012; Bourin et al., 2013; Gugjoo, Fazili et al. 2018; Gugjoo, Makhdoomi et al. 2019; Gugjoo, Fazili et al. 2020). Adipose-derived stromal cells (ADSCs) present in AdSVF can be maintained and expanded *in vitro* without losing their differentiation potential for long periods (Mazini et al. 2019).

AdMSCs are now being explored and utilized with promising prospects in clinical trials for their beneficial values in regenerative medicine and possessing therapeutic potential in human and veterinary medicine (Amarpal et al. 2013; Pieri et al. 2019; Rajabzadeh et al. 2019; Si et al. 2019; Al-Ghadban and Bunnell 2020; Laloze et al. 2021). However, several challenges have to be addressed related to the safety and efficacy of AdMSCs in clinical practice. This requires establishing strict quality control measures and safety tests at stages such as isolation and culture, cryopreservation, thawing, and expansion (Luo et al. 2021). For stromal cells, endothelial cells, and hematopoietic cell lineages, AdSVF has become an easily accessible source (Gentile and Cervelli 2018; Stefanis et al. 2019; Sun et al. 2019). To harvest, isolate and culture AdMSCs, it takes at least 2-3 weeks to get sufficient cell concentration. To preclude such a step, AdSVF harbours AdMSCs along with other growth factors and reduces the risk of culture period contamination. Therefore, it is a safe and cost-effective strategy (Kim et al. 2012; Gugjoo, Amarpal, et al. 2020). In addition, AdSVF offers a source full of regeneration potential to the extent of patient side utilization with little required maneuvering. However, despite the advantages, veterinary therapeutic research on AdSVF remains limited compared to cultured mesenchymal stem cells.

Although several studies have been conducted to evaluate the safety and efficacy of AdSVF in veterinary patients, the data available from these studies are scattered, limiting us from reaching a consensus on their clinical utility. Therefore, this review aims to evaluate the therapeutic potential of adipose-derived AdSVF in veterinary clinical practice with a particular focus on its applications in bone healing and regeneration. This is the first comprehensive review that gives an overall perspective of the therapeutic prospects of AdSVF in veterinary practice.

## 2. Why stromal vascular fraction?

Mesenchymal stem cell (MSCs) populations isolated from different tissues possess unique characteristics with varying proliferation and differentiation potential. Therefore, these differences should be considered while planning for specific clinical use (Fathi and Farahzadi 2016). Bone marrow harbours MSCs in a very limited concentration (0.01% to 0.001%) (Bhat et al. 2021; Dar et al. 2021). The mononuclear cell (MNC) fraction harvested contains MSCs that are culture expanded. BM-MSCs have been widely evaluated for regenerative therapeutics in varied conditions. But harvesting BM is very cumbersome and painful and increases the chances of infection (Sun et al. 2019). Contrarily, adipose tissue as a source of MSCs is gaining importance in regenerative stem cell therapy due to the higher concentration of MSCs (100–1000 times) as compared to bone marrow (BM-MSCs) (Nakao et al. 2010; Dar et al. 2021; Hendawy et al. 2021). In addition, the AdSVF contains angiogenic stem cells that promote vascular ingrowth and outgrowth (Wu et al. 2019). It also has a heterogeneous group of cells comprising stromal, endothelial, and hematopoietic cell lineages that spontaneously form robust and functional vasculatures (Sun et al. 2019).

Even the addition of growth factors like platelet-derived growth factor-BB (PDGF BB) enhances the osteogenic differentiation (calcium mineralization) of the AdMSCs as compared to the BM-MSCs (Hung et al. 2015). Compared to other sources of mesenchymal stromal/stem cells, subcutaneous adipose tissue contains pre-adipocyte cells commonly seen in the adventitia of blood vessels. These cells present in both AdSVF (freshly isolated cells) and the adherent fraction of AdMSCs have a significant role in managing chronic inflammation mainly due to their anti-inflammatory potential. However, the true potential of pre-adipocyte cells has not yet been fully understood (Baptista 2020).

Recent studies have used freshly isolated AdSVF cells instead of cultured AdMSCs (Upchurch et al. 2016; Kemilew et al. 2019). Expanding AdSVF cells to AdMSCs alters the phenotype, thereby reducing the differentiation (adipogenic and chondrogenic) potential. Therefore, the freshly isolated AdSVF cells have better regenerative capacity than cultured AdMSCs (Lee et al. 2014). The AdMSCs present in the AdSVF has the ability to attach and proliferate on calcium phosphate scaffolds. This process is followed by osteogenic differentiation, thereby favouring bone healing (Overman et al. 2013).

## 3. Preparation of adipose-derived stromal vascular fraction

**Figure 1.**
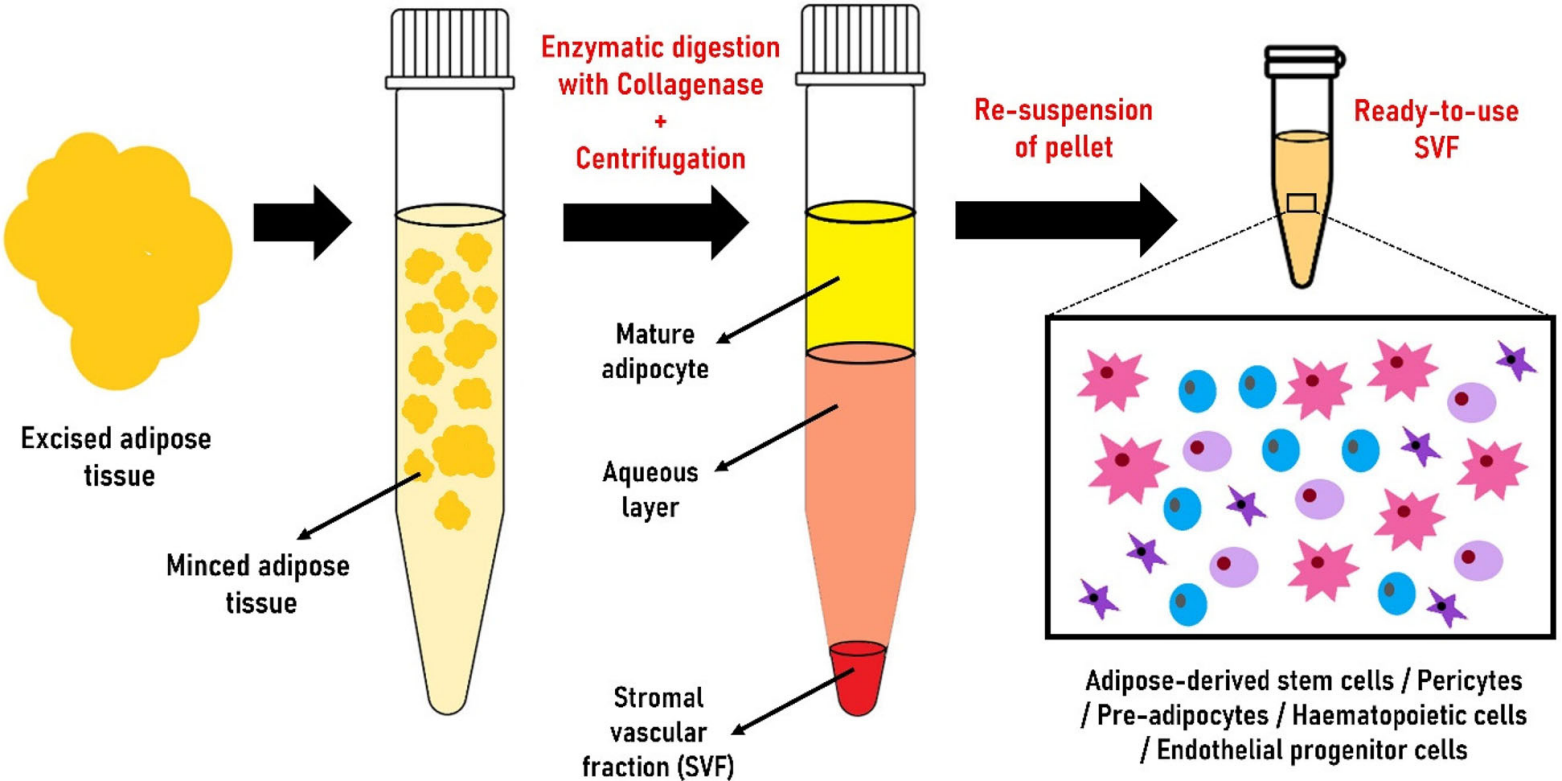
Steps involved in producing adipose-derived stromal vascular fraction (AdSVF) from adipose tissue collected from the fat depots using the enzymatic technique.

AdSVF is prepared from the subcutaneous adipose tissue collected from different parts of the animal (tail base in horses and inguinal region in dogs and cats) (Marx et al. 2015). AdSVF is commonly isolated by enzymatic or non-enzymatic (explant) techniques (Bora and Majumdar 2017; Senesi et al. 2019; Gugjoo, Amarpal et al. 2020). The enzymatic technique is widely used to isolate AdSVF from adipose tissue by digestion with collagenase (Figure 1) (Bora and Majumdar 2017; Gugjoo, Amarpal, et al. 2020; Sharun, Dhama, et al. 2021). It is considered the gold standard method for AdSVF isolation (Senesi et al. 2019). The enzymatic digestion will separate the contents into two distinct phases: upper mature adipocytes fraction (floats) and lower aqueous fraction (contains cellular fraction) (Bora and Majumdar 2017). The separation into different fractions can be enhanced by gravity-based phase separation (centrifugation). In addition, filtration can be performed to capture the required cell types based on size (SundarRaj et al. 2015; Bora and Majumdar 2017). Studies suggested that freshly isolated AdSVF, which is highly packed with adipose-derived stem cells, has a great potential to promote bone regeneration when combined with bone substitutes (Prins et al. 2016). Erythrocytes are one of the major contaminants present in the AdSVF pellet giving a reddish colour. It can be lysed using an RBC lysis buffer to isolate pure AdSVF cells (Riis et al. 2015). The cellular yield of equine AdSVF was previously evaluated using different concentrations of type I collagenase solution (0.1%, 0.05%, and 0.025%). Digestion of supragluteal subcutaneous adipose tissue using 0.1% type I collagenase solution yielded the highest number of nucleated cells (Duan and Lopez 2018).

The enzymatic method of isolating AdSVF is time-consuming (van Dongen et al. 2019). The non-enzymatic technique of AdSVF isolation involves mechanical agitation that breaks down the adipose tissue releasing stromal cells (Aronowitz et al. 2015; Bora and Majumdar 2017). AdSVF isolated using mechanical methods is equally safe and has advantages like low cost and less time-consuming (Aronowitz et al. 2015; Senesi et al. 2019). However, it has fewer progenitor cells and a high concentration of mononuclear cells. In addition, the cellular yield from mechanical techniques is lower than the enzymatic methods since mechanical action alone cannot release the tightly bound adipose tissue (Aronowitz et al. 2015; Tiryaki et al. 2020). On the contrary, a more robust final AdSVF product is generated since the mechanical technique preserves the extracellular matrix niche (Tiryaki et al. 2020; Gugjoo, Amarpal, et al. 2020). Another protocol used for AdSVF isolation is based on sonication-mediated cavitation. It is a safe, rapid, and cost-effective method that requires further validation (Amirkhani et al. 2016). Another non-enzymatic AdSVF isolation technique is the fractionation of adipose tissue procedure (FAT). This method can isolate AdSVF within 10-12 min, facilitating intraoperative isolation and rapid implantation (van Dongen et al. 2019). The viability of the AdSVF cells and cellular yield can be estimated using a trypan blue exclusion test and a haemocytometer (Hendawy et al. 2021).

The adipose tissue harvesting site should be selected based on different factors such as patient factors and biological factors. The viable cells per gram of adipose tissue obtained after processing mainly depend on the source or site of collection. The yield of viable cells obtained from the adipose tissue collected from the falciform location was significantly lower than the tissues collected at the inguinal and thoracic wall locations in dogs (Astor et al. 2013). Although breed size and body condition score did not affect the yield of viable cells, the age of the animal had a significant impact on the cellular yield (Astor et al. 2013). Therefore, such factors should be considered before the collection of adipose tissue.

The cellular components of AdSVF can be resuspended in platelet-rich plasma (PRP), phosphate-buffered saline (PBS), or 0.9% sodium chloride (saline) to produce a ready-to-use solution (Bukowska et al. 2021). In addition, AdSVF can be expanded *in vitro* to obtain AdSVF-derived mesenchymal stem cells that differentiate into diverse lineages of cells (Han et al. 2015). The quality and quantity of AdSVF is directly dependent on the harvesting site. Hendawy et al. (2021) compared the quality and quantity of AdSVF isolated from the subcutaneous abdominal, peri-ovarian, and falciform ligament fat depots. The study identified peri-ovarian site as the best (highest viability, cellular yield, and expression of AdMSCs surface markers) adipose tissue sampling site in dogs (Hendawy et al. 2021).

According to the joint statement of the International Society for Cellular Therapy (ISCT) and the International Federation for Adipose Therapeutics (IFATS), AdSVF cells can be phenotypically identified using the markers: CD45-CD235a-CD31-CD34+. In addition, further characterization can be performed using the surface antigens: CD13, CD73, CD90, and CD105 (Bourin et al., 2013). The cellular components of AdSVF can get further differentiated into endothelial cells or adipocyte-like cells based on the medium used. The differentiation of AdSVF cells towards endothelium is stimulated by the absence of adipogenic factors and the presence of serum (Balwierz et al. 2008).

## 4. Composition of stromal vascular fraction

**Figure 2.**
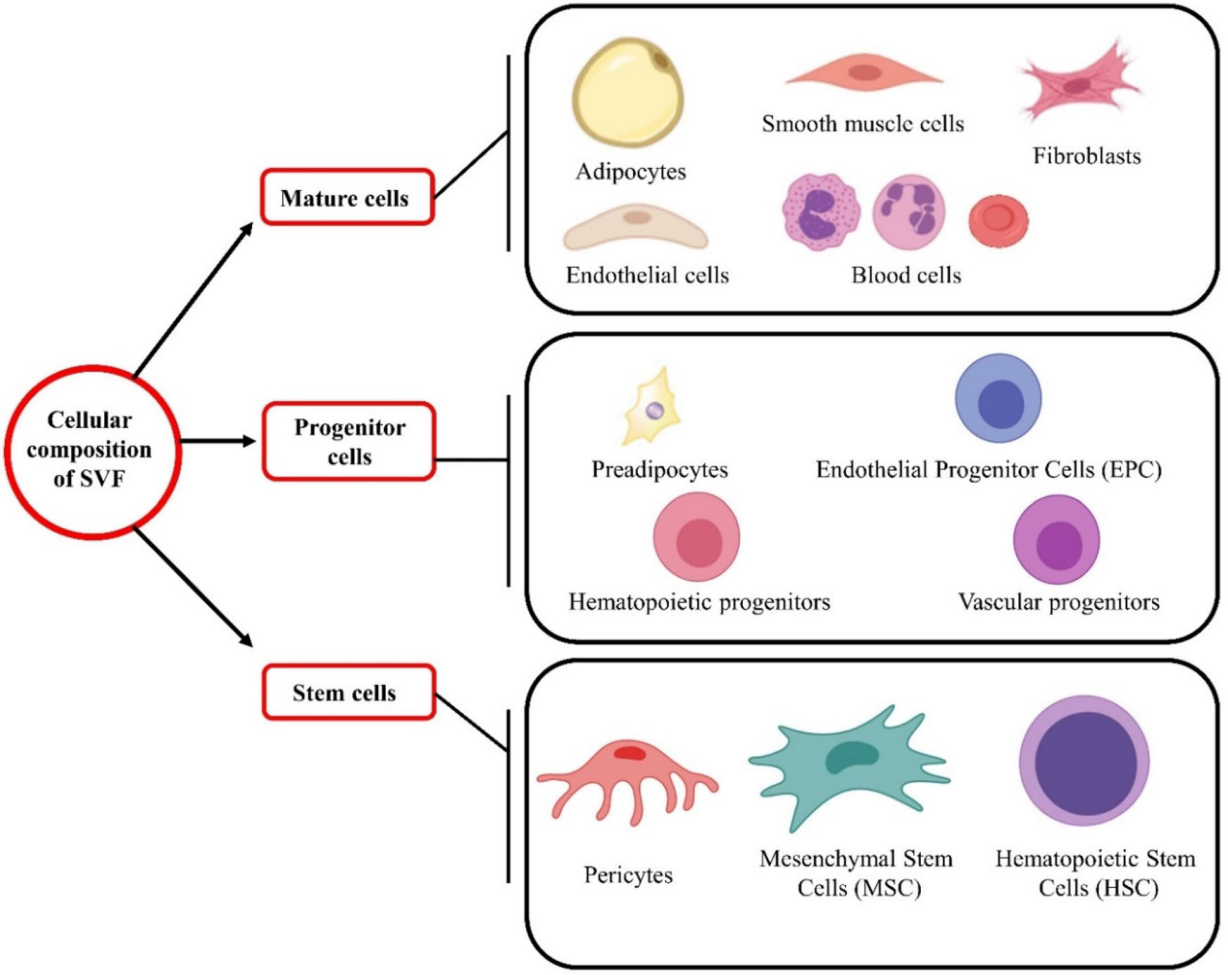
The major and minor cellular components present in the stromal vascular fraction: mature cells (adipocytes, smooth muscle cells, fibroblasts, endothelial; cells, and blood cells), progenitor cells (pre-adipocytes, endothelial progenitor cells, hematopoietic progenitors, and vascular progenitors), and stem cells (pericytes, mesenchymal stem cells, and hematopoietic stem cells).

The cellular constituents of AdSVF are illustrated in Figure 2. AdSVF contains variable cellular fractions depending on the species, fat source, age, gender, and physiological phase of the donor (Metcalf et al. 2016; Dar et al. 2021). One of the studies demonstrated that AdSVF composes mainly of AdMSCs (15–30%) along with other cellular components like immune cells (25–45%), endothelial cells (10–20%), and pericytes (3–5%) (Bourin et al., 2013). Another study reported hematopoietic stem cells (2%), adipose-derived stem cells (2–5%), pre/endothelial cells (7%), pericytes/smooth muscle cells (2%), fibroblasts (47%), and finally, other cells like macrophages, and other blood cells (33%) (Figure 2) (Folgiero et al. 2010). The number of stem cells present in the AdSVF can vary depending on several factors. The number of nucleated cells in the adipose tissue ranges from 500,000 to 2,000,000 cells/g. Among these nucleated cells, 1- 10% are AdMSCs. Hence, the number of AdMSCs present in adipose tissue will range from 5000 to 200,000 stem cells/g (Baer and Geiger 2012; Dar et al. 2021). The composition of the AdSVF, as well as the proliferation rate and differentiation capacity of the AdSVF cells, depends on factors such as animal species, age, type (brown or white) and anatomical location (subcutaneous or visceral) of adipose tissue, type of surgical procedure, method of cell separation, culturing conditions, culture medium, exposure to plastic, and plating density (Gentile et al. 2012; Gugjoo, Amarpal, et al. 2020).

Various studies suggest that the AdSVF contains several growth factors at high concentrations like hepatocyte growth factor (HGF), transforming growth factor-beta (TGF- β), vascular endothelial growth factor (VEGF), placental growth factor (PGF), and moderate concentrations of angiopoietin (Ang-1 and Ang-2), and fibroblast growth factor (FGF-2) (Gimble et al. 2007; Brown 2018; Stefanis et al. 2019). Among these, HGF plays a significant role in embryonic organ development and wound healing in adults. Furthermore, VEGF induces the growth of new blood vessels, and PGF also plays a major role in angiogenesis and vasculogenesis (Brown 2018). At the same time, TGF-β controls cellular proliferation and differentiation. In addition, FGF-2 promotes wound healing and angiogenesis, whereas Ang-1 and Ang-2 are involved in angiogenesis and the formation of blood vessels (Brown 2018).

Adipose tissue is considered an alternative source of MSCs. AdSVF contains multipotent progenitor/stem cells with chondrogenic, osteogenic, and adipogenic differentiation potential (Murphy et al. 2013). The MSCs present in the AdSVF can differentiate into several specific cell types like osteoblasts, chondrocytes, myoblasts, and fibroblasts, which have wide application in regenerative medicine (Mizuno et al. 2012). One of the major prerequisites for successful tissue regeneration is adequate vascularization. Several strategies have been evaluated to improve the vascularization of tissue-engineered grafts (Wu et al. 2019). Some of these strategies involve using a pre-vascularized graft or microvascular fragments instead of conventional grafts that depend exclusively on the host tissue for vascularization, angiogenesis, and vasculogenesis. These grafts contribute to the vascularization process (angiogenesis and vasculogenesis) from the graft to the host, further enhancing the process (Wu et al. 2019). AdSVF being a rich source of VEGF and a variety of progenitor cells may contribute to the vascularisation process of bone grafts.

## 5. Therapeutic potential of stromal vascular fraction

**Figure 3.**
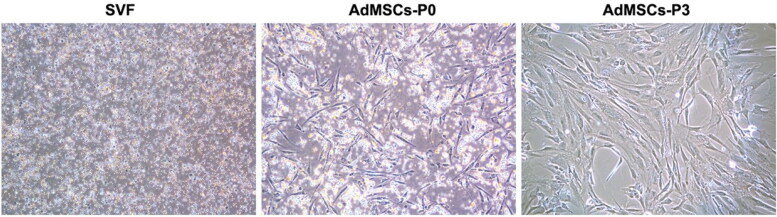
Cell morphologies of freshly isolated stromal vascular fraction (AdSVF) and cultured adipose-derived mesenchymal stem cells (AdMSCs) at passages 0 and 3. Reproduced from Zhou et al. (2017) under Creative Commons Attribution 4.0 International License (https://creativecommons.org/licenses/by/4.0/).

**Figure 4.**
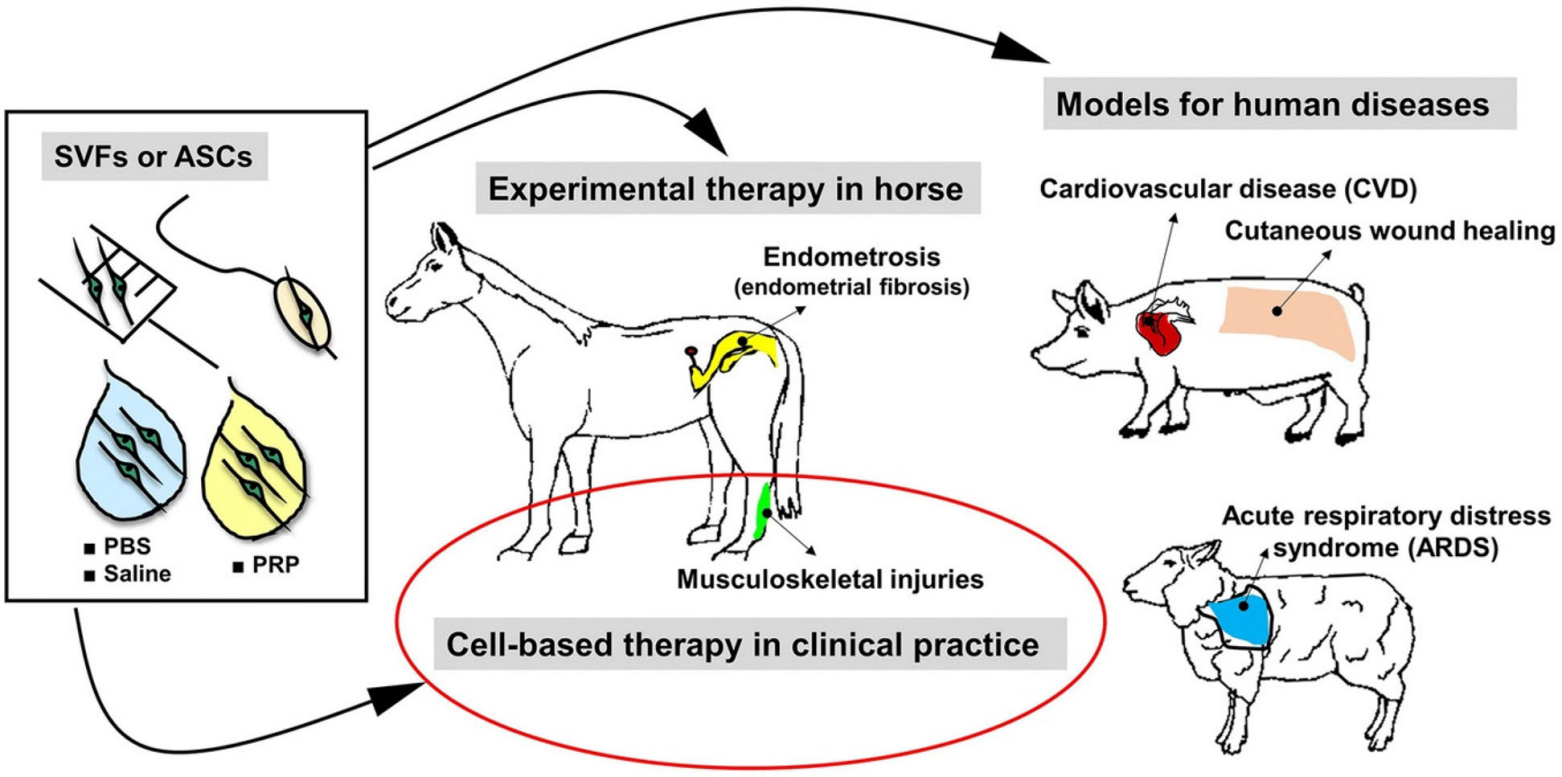
Applications of stromal vascular fraction (AdSVF) or adipose-derived stem cells (ASCs) in translational research involving pigs, horse, and sheep models and veterinary practice. ASCs/SVFs can be suspended in either platelet-rich plasma (PRP), phosphate-buffered saline (PBS), or 0.9% sodium chloride (saline). The suspended cells can be administered via different routes, including intra-lesional, intravenous injections (musculoskeletal injury treatment, cutaneous wound healing, acute respiratory distress syndrome), intracoronary delivery using balloon angioplasty catheter (cardiovascular disease), or insemination catheter (endometriosis). Reproduced from Bukowska et al. (2021) under Creative Commons Attribution 4.0 International License (https://creativecommons.org/licenses/by/4.0/).

Regenerative medicine essentially employs a cellular component. Among various cells, stem cells or their products are increasingly being evaluated for veterinary applications (Ribitsch et al. 2010; Amarpal et al. 2013; Pieri et al. 2019; Rajabzadeh et al. 2019; Russell et al. 2020; Voga et al. 2020; Kumar et al. 2021; Prządka et al. 2021) in dogs (Gugjoo, Amarpal, et al. 2019), cattle/buffalo (Gugjoo, Fazili, et al. 2018; Hill et al. 2019), cat (Gugjoo, Fazili, et al. 2020; Quimby and Borjesson 2018), horse (Lopez and Jarazo 2015; Gugjoo, Makhdoomi, et al. 2019), sheep (Gugjoo 2018; Dar et al. 2021) and goat (Gugjoo, Fazili, et al. 2020). As adipose tissue originates from the mesodermal layer, AdSVF can be directly applied, thereby considered a feasible patient side treatment option (Figure 3). Adipose-derived stromal cells can differentiate into osteogenic, adipogenic, chondrogenic, and myogenic lineages (Gimble et al. 2007). The therapeutic potential of AdSVF has been previously evaluated in different animal models (Figure 4). Many studies have already explored the potential of these cells for osteogenic differentiation in animal models using various scaffolds and biomaterials as cellular carriers (Levi et al. 2010; Phipps et al. 2011). These cells can be easily differentiated toward the osteogenic cell lineage and expanded and cultured in large amounts for tissue engineering purposes (Almubarak et al. 2016). AdSVF also contains other cell types in addition to MSCs. Therefore, the allogeneic use of AdSVF currently remains controversial (Bora and Majumdar 2017).

### 5.1. Stromal vascular fraction for osteoarthritis

Osteoarthritis is a chronic joint disease that occurs secondarily to developmental orthopaedic diseases such as hip/elbow dysplasia, cranial cruciate ligament disease, and patellar dislocation (Ivanovska et al. 2022). Secondary osteoarthritis is the common type of osteoarthritis seen in dogs. It mainly affects the stifle, hip, and elbow joints (Pettitt and German 2015). The therapeutic strategies commonly used for managing canine osteoarthritis mostly focus on controlling the pain and inflammation associated with the disease progression and are not directed at disease modification (Brondeel et al. 2021). MSCs are already being evaluated for managing osteoarthritis due to their ability to restore cartilage defects (Brondeel et al. 2021; Ivanovska et al. 2022). In addition, regenerative medicine involving cellular therapy is increasingly used as a common mode of treating osteoarthritis (Gugjoo, Chandra et al. 2018; Gugjoo, Fazili, et al. 2019). AdSVF has been evaluated as therapeutics either alone or more recently, along with the platelet-rich plasma (PRP). Available literature indicates that AdSVF can safely be administered in dogs with hip osteoarthritis (intra-articular and intravenous administration) (Upchurch et al. 2016). However, clinical data on stifle and elbow osteoarthritis are lacking, limiting our ability to reach a conclusion.

Osteochondral defects created in medial condyles and trochlear grooves (5 mm x 3 mm) treated with AdSVF (5 × 10^6^ cells) loaded onto the acellular collagen type I/III scaffold had led to the regeneration of collagen type II, hyaline-like cartilage. The regenerated tissue had higher elastic moduli with glycosaminoglycan content comparable to the native tissue cartilage. The healing was comparable to that led by the implantation of AD-MSCs (5 × 10^5^ cells) and was evaluated through macroscopy, immunohistochemistry, biomechanical analysis, micro-CT analysis, and biochemistry (Jurgens et al. 2013). In the case of hip dysplasia, AdSVF (0.2 to 0.8 × 10^6^) transplantation at acupoints improved the range of motion, lameness at the trot, and pain on manipulation of the joints after 30 days (Marx et al. 2014). AdSVF being rich in growth factors in addition to the cells might have led to improved healing of osteochondral defects (Kazemi et al. 2017). However, the implantation of AdSVF (16.3 × 10^6^) or BM-MSCs (10.5 × 10^6^) failed to improve the healing of middle carpal joint osteochondral defect, except for the PGE2 levels after 70 days period. In the case of AdSVF transplanted joints, the tumor necrosis factor-α (TNF-α) level was increased in the synovial fluid (Frisbie et al. 2009). Similarly, another study demonstrated that AdSVF or AdSVF loaded on the poly L-lactide-co-glycolide (PLGA) nanofiber scaffold had reported no significant improvement in function, cartilage biochemical composition, or histology. Simultaneously AdSVF loaded onto the PLGA had led to adverse results. In contrast, leukocyte poor PRP led to the better healing of the chondral defects in the knee joint in dogs during six months follow-up (Franklin et al. 2018). The therapeutic benefits of AdSVF in affected joints are mediated through the paracrine anti-inflammatory and immune-modulatory mechanisms (Andia et al. 2019).

Apart from the use of AdSVF as monotherapy, the combined use of AdSVF and PRP is also commonly reported in the management of osteoarthritis as well as articular cartilage injury treatment (Van Pham et al. 2013; Upchurch et al. 2016). In an experimental study, chondral defects treated with low laser irradiated AdSVF along with PRP resulted in cartilage regeneration and restoration of the chondral histomorphological picture over six months. In contrast, the control defects deteriorated over time (Abdallah et al. 2015). Intra-articular injection of AdSVF and PRP improved the Canine Brief Pain Inventory (CBPI) score and peak vertical force (PVF) in dogs with osteoarthritis of the hip joints (Upchurch et al. 2016). Intravenous administration of allogenic AdSVF increased the VEGF levels in serum of dogs with spine degenerative joint disease. The overexpression of VEGF indicated the proangiogenic effects of AdSVF that stimulated regenerative processes in the damaged tissues (Kemilew et al. 2019). In addition, the injected AdSVF cells induce a cascade of structural and molecular events due to the interactions between AdSVF and infrapatellar fat pad that promotes the regeneration of damaged tissues (Lapuente et al. 2020). Intra-articular injection of autologous AdSVF along with hyaluronic acid (HA) had therapeutic efficacy in preventing the progression of osteoarthritis and promoting cartilage regeneration in sheep model (anterior cruciate ligament transection and medial meniscectomy). However, it was lesser than that of autologous AdMSC combined with HA (Lv et al. 2018).

### 5.2. Stromal vascular fraction for wound healing

AdSVF is composed of different cell populations, and most are competent in influencing the wound microenvironment (Fraser et al. 2014). For example, in experimentally induced full-thickness burn wounds of rats, AdSVF, and ADSCs, paracrine secretion of PDGF and bFGF contributed to increased fibrin and fibroblasts (Kim et al. 2009). In addition, the cytokines released by AdSVF cells can change the macrophage activation profile from classic to regulatory. This improves the wound healing profile (Gourevitch et al. 2014). AdSVF promotes wound healing by regulating gene expression and enhancing the function of endothelial cells and fibroblasts (Bi et al. 2019). The combined use of AdSVF and platelet-rich plasma has an additive stimulatory effect that supports angiogenesis, thereby accelerating the wound healing process (Karina et al., 2019). Therefore, such a combination can be considered an alternative to AdMSC treatment.

The therapeutic potential of AdSVF in wound healing could be attributable to the secretion of chemokines, epidermal growth factor, epithelialization growth factors, neutrophil-activating protein-2 (NAP-2 or CXCL7), and stromal cell-derived factor (SDF-1 or CXCL12) (Chae et al. 2017). An injectable extracellular matrix-AdSVF gel is an attractive therapeutic strategy for enhancing wound healing (Sun et al. 2017). The extracellular matrix-AdSVF gel increased the expression of angiogenic factors such as vascular endothelial growth factor and basic fibroblast growth factor. Therefore, the potent angiogenic effects exerted by AdSVF might have contributed to the improvement of wound healing (Sun et al. 2017). In addition, AdSVF was also found to be effective for managing deep partial-thickness burn wounds in rats. It induced healing by reducing inflammation of the burn wound and increasing fibroblastic activity, proliferation, and vascularization (Atalay et al. 2014). Implantation of AdSVF also accelerates re-epithelialization and wound closure (Chae et al. 2017). In another study, AdSVF was found to promote fibroblast migration and cellular viability in a hyperglycaemic microenvironment with the help of wound healing cytokines. This indicates therapeutic potential in diabetic wound management (Tan et al. 2018). Intradermal injection of AdSVF was also found to enhance epithelialization and angiogenesis in full-thickness cutaneous wounds in rats (Karagergou et al. 2018). Therefore, AdSVF accelerates wound healing by enhancing angiogenesis and neovascularization (Andia et al. 2019).

Apart from direct skin wound healing, AdSVF appears promising in anorectal fistula commonly seen in dogs. For example, in a porcine model of mechanically induced anorectal fistula, transplantation of AdSVF led to the complete healing of the fistula in two weeks (Dryden et al. 2017). Furthermore, AdSVF appears promising in preventing gastrointestinal fistula tracts following gastrointestinal surgery, as demonstrated in rabbits (Aldaqal et al. 2015).

### 5.3. Stromal vascular fraction for promoting bone healing and regeneration

AdMSCs have broad applications in bone tissue engineering due to their *in vivo* osteogenic potential. They also demonstrate significant angiogenic potential, making them suitable for augmenting bone healing (Kim et al. 2012). AdSVF is currently used to manage various orthopaedic conditions in clinical settings. It has superior therapeutic potential for regenerating bone, cartilage, and tendons. Hence AdSVF has wide application in regenerative medicine and is used for treating osteonecrosis (Pak et al. 2017). The AdSVF has several clinical applications and can be used for managing bone diseases that involve loss of bone, osteonecrosis, and oncologic bone resections (Roato et al. 2018). Autologous AdSVF cells combined with bone substitutes and other biological factors can be considered an alternative to conventional bone grafting techniques (Najman et al. 2016). Freshly isolated AdSVF exhibited promising osteogenic and vasculogenic capacity (Najman et al. 2016). Besides potentiating osteogenesis, AdSVF diminishes the possibility of osteonecrosis at the bone ends (Toplu et al. 2017).

In a study by Pak, autologous AdSVF exhibited bone regeneration potential in man that was further used for managing osteonecrosis of the femoral head (Pak 2011). By modifying the media used for AdSVF collection and storage, we can induce osteogenic differentiation of AdSVF cells. Such differentiated AdSVF cells exhibit superior bone healing capacity compared to undifferentiated AdSVF cells (Kim et al. 2012). In addition, AdSVF contains a large number of CD34 + CD45" cells that can stimulate angiogenesis and play a major role in the neovascularization processes to promote the healing of ischemic tissues (Madonna and De Caterina 2008).

Even though AdSVF and ASC exhibit equal *in vitro* osteogenic differentiation potential, the AdSVF construct was found to possess superior bone-regenerative capacity compared to the ASC construct upon implantation in the rat model of the femoral bone defect (Zhang et al. 2018). AdSVF also exhibited better osteoinductive potential than ASCs when it was plated on a xenohybrid bone scaffold in an osteogenic medium (Roato et al. 2018). Furthermore, freshly isolated AdSVF expressed bone-related and endothelial-related genes, making it an excellent therapeutic candidate for managing bone defects (Najman et al. 2016). The superior osteogenic differentiation potential of AdSVF was also associated with the distinct differences in immunoregulatory effects from ASCs (Zhang et al. 2018). On the contrary, in another study, ASC-loaded scaffolds produced greater bone volume and coverage area than the AdSVF-loaded scaffolds (but not statistically significant) in a murine model of critical-sized cranial defects (Nyberg et al. 2019).

MSC-rich AdSVF was also found to increase bone healing in an experimental zygomatic bone defect rat model. This technique can further replace the clinical use of bone grafts and flaps (Toplu et al. 2017). Intraoperative implantation of freshly isolated AdSVF cells without *in vitro* expansion is currently being used to treat bone defects (Aslan et al. 2006; Evans et al. 2007). This technique is simple, time and cost-effective, minimally invasive, and the process of isolation to implantation will take only a few hours (Müller et al. 2010; Coelho et al. 2012).

In addition to progenitor cells (AdMSCs), AdSVF contains several growth factors such as TGF-β, IGF1, FGF2 and PDGF that accelerate the bone healing process (Sananta et al. 2022). TGF-β is essential for the maintenance and expansion of MSCs, maintenance and differentiation of osteoblasts, and osteoprogenitor cell proliferation (Chen et al. 2012; Sananta et al. 2022). Administration of AdSVF enhanced the healing process in the murine bone defect model characterized by an increased level of TGF- β1 (Sananta et al. 2022).

Autologous AdSVF has also been combined with calcium phosphate ceramics to promote bone regeneration. Implantation of calcium phosphate ceramic seeded with freshly isolated AdSVF in the maxillary sinus floor elevation model in man was an effective and safe bone regeneration technique (Prins et al. 2016). Adipose-derived AdSVF was also found to enhance the remodelling of devitalized hypertrophic cartilage to bone tissue in the rat calvarial defect (4 mm diameter) model (Todorov et al. 2016). However, in a study by Thery et al. (2015) to investigate the osteogenic potential of AdSVF, it was found that the combination of AdSVF and BCP was insufficient to promote bone formation. Hence, it was opined that an osteoinductive factor should be included to promote the differentiation of osteoblasts, thereby supporting bone tissue formation (Müller et al. 2010).

### 5.4. Stromal vascular fraction for tendon healing

In tendons, healing is hampered due to its lack of direct blood supply and the limited concentration of less active multiplying tenocytes. Therefore, treating tendon or ligament injuries is a Herculean task as regenerative medicine is being evaluated (Gugjoo, Fazili, et al. 2018; Gugjoo, Makhdoomi, et al. 2019; Gugjoo, Fazili, et al. 2020). In order to improve healing, various growth factors along with the cells may be incorporated for enhanced scar- and adhesion-less healing. Co-culture of AdSVF with tenocytes has been demonstrated to enhance gene expression for insulin‐like growth factor‐1 (IGF‐1), stromal cell‐derived factor‐1α (SDF‐1α), transforming growth factor‐β1 (TGF‐β1) and TGF‐β3. Such an enhancement has been significantly higher with AdSVF compared to AdMSCs (Polly et al. 2019). In the horse, regarding superficial digital flexor tendonitis (collagenase-induced) treatment with AD-AdSVF (three doses at 40-53 hr of 13.83 ± 3.41 × 106 cells) significantly improved tendon fiber architecture with reductions in vascularity, inflammatory cell infiltrates, and collagen type III formation. Furthermore, tendon fiber density and alignment were also improved. However, gene expression analysis of collagen type I and type III failed to show any difference between control and cell treatment at six weeks (Nixon et al. 2008). Similarly, implantation of autologous adipose micrografts along with AdSVF in the sheep model had improved common calcaneal tendinopathy. Tendon diameter, fiber orientation score, fiber edema score, infiltrative-inflammatory process, and necrosis score showed improvement compared to the control (Piccionello et al. 2021).

### 5.5. Miscellaneous applications

In the generation of tissue-engineered bladders, bladder vascularization is an important aspect. AdSVF possesses all the requisite cell populations for promoting cell repair in tissue engineering (Leblanc et al. 2012; Zhou et al. 2016). The angiogenic factors viz. VEGF, PDGF-BB, and bFGF catalyze and enhance neovascularization in tissue-engineered bladders in man (Zhou et al. 2013). AdSVF is similar to AdMSCs and is capable of intrinsic angiogenesis (Nunes et al. 2013). Zhao et al. (2019) reported statistically significant advantages of AdSVF over AdMSCs. AdSVF and bladder acellular matrix (BAM) combination enhances neovascularization. Apart from this, AdSVF has also shown better safety and tolerance when used along with BAM than BAM alone (Mizuno et al. 2012; Bora and Majumdar 2017).

Two different MSC products are in the pipeline as a novel therapy for treating chronic kidney disease in felines: AdSVF (non-expanded) and AdMSCs expanded in culture (Quimby et al. 2016). Intrarenal autologous MSC injections for feline CKD were safely tolerated and improved renal function significantly (Quimby et al. 2011). Both bone marrow MSCs and AdMSCs showed highly significant kidney protective effects, viz. decreased fibrosis, intrarenal inflammatory infiltrate, and sclerosis of the glomerulus (Lee et al. 2010; Villanueva et al. 2011). In addition, overall health and weight, BUN, creatinine, BP, and haematocrit showed marked improvement. Various routes of administration in repeated doses, viz. subcapsular, IV, or intraparenchymal, are all effective (Semedo et al. 2009; Lee et al. 2010). Furthermore, administration of AdSVF attenuated acute rejection following organ donation in the circulatory death renal transplantation model in rats by enhancing indoleamine 2, 3-dioxygenase expression increasing regulatory T cells ratio (Wang et al. 2021).

Because of the multipotent nature of AdSVF, it was attempted to be used to treat traumatic brain injury in animal models. AdSVF administered soon after a traumatic brain injury could palliate and forestall motor skills and memory deficits that would otherwise occur in the absence of AdSVF. This was substantiated by the Rotarod test and Morris water maze (MWM) test (Berman et al. 2018). In addition, tail vein administered AdSVF within 4 hours of traumatic brain injury can increase the success of therapy as the injury initially alters the permeability of the blood-brain barrier (Beaumont et al. 2000). AdSVF loaded with the silicone rubber conduit improves rat sciatic nerve injury in diabetic (Mohammadi et al. 2015). The therapeutic potential of AdSVF derived from omental adipose tissue was evaluated in a rat sciatic nerve transection model by loading into a vein graft (Mohammadi et al. 2012). The findings indicate that AdSVF can be considered as an ideal candidate for peripheral nerve regeneration since it facilitates the functional recovery of sciatic nerve injury (Mohammadi et al. 2012).

In treating human patients with scarred vocal folds, AdSVF is a new hope. It enhances the healing process and reduces granuloma, fibrosis, and inflammation (Mattei et al. 2017). Autologous AdSVF injection in scarred vocal folds was found to be safe and tolerable in humans (Mattei et al. 2020). In addition, the viscoelasticity and vibration amplitude was restored (Hiwatashi et al. 2017). Furthermore, the M2 phenotype macrophages and regulatory T cells in AdSVF express a high level of immunosuppressive cytokines that contribute to immunomodulatory effects. Similar studies must be conducted on veterinary patients to establish the therapeutic potential of AdSVF in vocal fold-associated pathologies. In rats with acute or chronic liver failure (ACLF), the AdSVF serves as a promising therapeutic agent. Ho et al. (2019) studied the therapeutic effects of CD 34+/CD34- AdSVF cells in hepatocyte co-transplantation. The co-transplantation of CD34+ AdSVF cells ensured quick recovery from liver fibrosis and biliary ductular proliferation compared with CD34- AdSVF cells. Even in rat testicular degeneration, AdSVF appears promising (Gao et al. 2016; Zhou et al. 2019) and thus, may be an ideal therapeutic strategy for animals utilized for breeding purpose having elite germplasm. In addition, it may offer a promising strategy for preventing graft rejection (Weltz et al. 2020).

## 6. Limitations

Science is advancing rapidly, and the progressive advancements make it difficult to keep up with it, especially for a clinician. Although AdSVF and AdMSCs have already established their therapeutic utility in various disorders and diseases, they possess all the disadvantages of cell-based therapeutics (Sharun, Pawde, et al. 2021). Some disadvantages, such as tumorigenic potential, pulmonary embolism, and inability to reach target organs, limit the spectrum of therapeutic use (Sharun, Pawde, et al. 2021). In addition, further efforts are required to optimize the sources of AdSVF and estimate the transplantation dose and delivery methods to standardize the therapeutic protocols (Devireddy et al. 2017; Kang and Park 2020). Specific standards or recommendations are not available that define the critical attributes of cell-based products derived from veterinary species (Devireddy et al. 2017). Studies must be conducted to determine the storage life of AdSVF, cryopreservation, and their reuse. Furthermore, large-scale manufacturing techniques have to be developed that ensures quality assurance and control following current Good Manufacturing Practices (Gimble et al. 2017).

Fat is also a common tissue affecting the endocrine functioning in the body. It specializes in acting as a storehouse for various chemicals, including endocrine-disrupting chemicals (EDCs). Accumulating such chemicals affects the mesenchymal stem cell properties of the adipose tissue (AD-MSCs). EDCs promote adipogenic differentiation of the AD-MSCs while simultaneously decreasing the osteogenic differentiation (Marycz, Kornicka, Basinska, et al. 2016; Marycz, Kornicka, Grzesiak, et al. 2016; Marycz, Kornicka, Marędziak, et al. 2016; Marycz et al. 2018; Marycz 2021). Additionally, EDCs promote pro-inflammatory cytokines and increase oxidative stress, lowering their activity (Pakzad et al. 2013; Ricciardi et al. 2012; Hayrapetyan et al. 2015; Bateman et al. 2016). Similarly, in equine metabolic syndrome (EMS), mitochondrial biogenesis and function impairments tend to affect MSCs osteogenesis (Marycz, Kornicka, Basinska, et al. 2016; Marycz, Kornicka, Grzesiak, et al. 2016; Marycz, Kornicka, Marędziak, et al. 2016). It has been recently proposed that AD-MSCs and hepatic stellate cells have a critical endocrine relationship that might be responsible for metabolic syndrome (Marycz 2021). Thus, AdSVF derived from adipose tissue may not be effective in such cases.

## Conclusion and prospects

AdSVF can be isolated from adipose tissue using enzymatic or non-enzymatic techniques. The enzymatic technique is widely used since it is considered as the gold standard method for AdSVF isolation. AdSVF isolation using mechanical methods is equally safe but has a lower cellular yield. The cellular components of AdSVF can be resuspended in PRP, PBS, or 0.9% sodium chloride to produce a ready-to-use solution.

Adipose-derived AdSVF has osteogenic, adipogenic, chondrogenic, and myogenic potential that can be used for treating a wide array of diseases and disorders. It is currently being used for managing various orthopaedic conditions in clinical settings and has the potential for regenerating bone, cartilage, and tendons. In addition, AdSVF can also be used to promote wound healing and be considered a therapeutic strategy for managing osteoarthritis and tendonitis. Furthermore, the anti-inflammatory activity and immunomodulatory potential of AdSVF can be utilized for managing immune-mediated and inflammatory diseases. The heterogeneous cellular composition of AdSVF (MSCs, pericytes, endothelial cells, fibroblasts, macrophages, and other immune cells) contributes to the broad therapeutic potential. The potential of AdSVF-based regenerative cell therapy is enormous and is currently at its infant stage. Future studies may widen the clinical utility of AdSVF further. However, in addition to *in vivo* studies, researchers should also focus on conducting large-scale, randomized clinical controlled trials in veterinary patients to establish its clinical utility.

Due to their relative ease of access and standardized laboratory procedures, ASCs have become the most popular sources of cells for stem cell-based therapy involving different tissues. However, the need for a sterile laboratory having culturing facilities limits the clinical utility of ASCs in veterinary practice. In addition, the isolation of ASCs from AdSVF is time-consuming since it requires an additional culture period. The interval from adipose tissue harvest to the injection of the final cellular product (ready-to-use AdSVF) is very short. Therefore, using freshly isolated AdSVF can preclude the additional culture period, reducing the risk of extensive cell contamination, thus making it a safe and cost-effective strategy. However, like all cell-based therapeutics, disadvantages such as pulmonary embolism and the inability to reach target organs limit their therapeutic use if administered intravenously. In addition, the prospects of allogeneic use of AdSVF remain controversial due to a lack of sufficient data. Finally, the lack of proper regulatory guidelines for isolation, characterization, and clinical use makes interpreting the results of clinical trials difficult. Although cell-free therapeutic strategies are replacing cell-based therapeutics, the ease of access and simplicity of the latter make it a promising treatment strategy for clinicians.
